# Association between Passive Smoking from the Mother and Pediatric Crohn’s Disease: A Japanese Multicenter Study

**DOI:** 10.3390/ijerph17082926

**Published:** 2020-04-23

**Authors:** Koji Uchiyama, Yasuo Haruyama, Hiromi Shiraishi, Kiyohiko Katahira, Daiki Abukawa, Takashi Ishige, Hitoshi Tajiri, Keiichi Uchida, Kan Uchiyama, Masakazu Washio, Erika Kobashi, Atsuko Maekawa, Kazushi Okamoto, Toshimi Sairenchi, Yuka Imamura, Shuji Ohhira, Akira Hata, Gen Kobashi

**Affiliations:** 1 Laboratory of International Environmental Health, Center for International Cooperation, Dokkyo Medical University, Tochigi 321-0293, Japan; 2 Department of Public Health, Dokkyo Medical University School of Medicine, Tochigi 321-0293, Japan; 3 Department of Health and Nutrition, University of Human Arts and Sciences, Saitama 339-8539, Japan; 4 Research Institute of Clinical and Social Pharmacy, Saitama 341-0035, Japan; 5 Department of Gastroenterology and Hepatology, Miyagi Children’s Hospital, Miyagi 989-3126, Japan; 6 Department of Pediatrics, Gunma University Graduate School of Medicine, Gunma 371-8511, Japan; 7 Department of Pediatrics, Osaka General Medical Center, Osaka 558-8558, Japan; 8 Department of Gastrointestinal and Pediatric Surgery, Mie University Graduate School of Medicine, Mie 514-8507, Japan; 9 Division of Gastroenterology and Hepatology, Department of Internal Medicine, The Jikei University Kashiwa Hospital, Chiba 277-8567, Japan; 10 St. Mary’s College, Fukuoka 830-8558, Japan; 11 Musashino University, School of Human Sciences, Tokyo 202-8585, Japan; 12 Department of Preventive Medicine, Nagoya University Graduate School of Medicine, Nagoya 466-8550, Japan; 13 Department of Epidemiology, Graduate School of Nursing and Health, Aichi Prefectural University, Aichi 480-1198, Japan; 14 Department of Public Health, Chiba University School of Medicine, Chiba 260-8670, Japan

**Keywords:** dose-response relationship, epidemiology, maternal smoking, passive smoking, pediatric Crohn’s disease

## Abstract

Smoking is a risk factor for adult-onset Crohn’s disease (CD). Although passive smoking from family members is a major concern, especially in pediatric CD, the number of existing epidemiological studies is limited. This multicenter case–control study aimed to assess the effects of familial smoking on pediatric CD. We examined 22 pediatric CD cases and 135 controls. The subjects’ mothers were given a self-administered questionnaire about family smoking before disease onset in the CD group or the corresponding period in the control group. Univariable logistic regression model was used to calculate odds ratios (ORs) and 95% confidence intervals (CIs), whereas dose–response relationship analyses were performed for more in-depth evaluations. Univariable analyses indicated that passive smoking from the mother (OR, 2.09; 95% CI, 0.61–7.10) was not a significant, but a candidate risk factor for developing pediatric CD. In contrast, the dose–response relationship analyses revealed that passive smoking from the mother (OR, 1.17; 95% CI, 1.04–1.31) was significantly associated with pediatric CD. Therefore, passive smoking from the mother may be predominantly associated with the development of pediatric CD. Further follow-up studies comprising environmental measurements of passive smoking exposure doses and genetic factors interaction analysis are necessary.

## 1. Introduction

Pediatric Crohn’s disease (CD), one of the main forms of pediatric inflammatory bowel disease (IBD), is often characterized by discontinuous mucosa-to-serosa inflammatory lesions. Genetics is known to play a key role during the early onset of IBD; however, the lack of complete penetrance of IBD among monozygotic twins [1,2], as well as its limited familial occurrence [3,4] and its increasing prevalence in Japan (age-standardized prevalence of child IBD 7.2/100,000 cases in 2013) [5]—Which was one of the traditionally low-incidence countries—Indicate that environmental factors also play an important role in the onset and development of these disorders.

Active cigarette smoking is one of the most consistently observed environmental influences on IBD; in fact, it is considered a risk factor for the development of CD [6,7]. Active smoking begun by age 15, though not allowed in many countries, was found to be associated with a future diagnosis of CD [8]. Conversely, the effects of fetal smoke exposure are still controversial: on the one hand, a case–control study reported a modest protective role of maternal smoking during pregnancy against pediatric CD [9]; on the other hand, other studies reported an association between maternal smoke exposure and the risk of young onset CD [8,10]. Furthermore, a recent gene–smoking interaction study in mice and humans suggested that some variants of nucleotide-binding oligomerization domain-containing protein 2 (NOD2) may interact with tobacco smoke, eventually modifying the risk for CD [11]. In contrast, although significant associations between NOD2 variants and CD have never been clarified in the Asian population, an increase in the risk of CD has been confirmed in Japanese smokers [12].

The association with familial passive smoking is a major concern in pediatric IBD. Although a previous meta-analysis failed to find any significant association between passive smoking and CD [13], pieces of evidence regarding passive smoking-related increases in the risk of pediatric or adolescent onset CD are gradually being accumulated [8,10,14,15,16]. Furthermore, a significant association between passive smoking and CD in the Japanese population has been recently identified [17]; however, the relationship between passive smoking and pediatric CD still needs to be clarified.

We conducted a multicenter case–control study in Japan to identify whether familial passive smoking is a risk factor for pediatric IBD. Since passive smoking is a potentially modifiable factor—Provided there is a widespread sensibilization and understanding of its potential risk for pediatric CD—This first report aimed to investigate the CD-related impact of familial passive smoking on the Japanese pediatric population.

## 2. Materials and Methods

### 2.1. Study Design and Materials

This multicenter case–control study, which involved five hospitals located in the eastern and western areas of Japan, included pediatric IBD patients attending the hospitals for clinical management from October 2010 to March 2016; in particular, the enrolled patients had been diagnosed with CD or ulcerative colitis (UC) at or below the age of 15 years. Diagnostic criteria were determined by the Crohn’s Disease Study Committee of the Japanese Society of Gastroenterology and the Research Committee of Ulcerative Colitis of the Japanese Ministry of Health and Welfare for CD and UC, respectively [18]. On the other hand, voluntary controls, including students and their related persons, were recruited from universities and colleges located in urban areas of eastern (i.e., Tokyo and Saitama) and western (i.e., Mie) Japan. Any subject in the control group who had a history of gastrointestinal disorder, autoimmune disorder, or intractable diseases was excluded.

The sample eventually comprised 94 pediatric IBD patients (i.e., CD, 30 [32%]; UC, 64 [68%]) and 164 controls. Besides, a questionnaire-based survey was simultaneously conducted in collaboration with the subjects’ mothers.

### 2.2. Questionnaires

A self-administered written questionnaire referring to the period from before the mother’s pregnancy to the date of the child’s IBD diagnosis—Or a corresponding period for controls—Was submitted to and filled by the mother of each subject. The questionnaire comprised five different categories of questions: (i) factors during the pre-pregnancy and post-partum periods (e.g., fertility treatment, disease during pregnancy, gestational age at birth, birth weight, hospitalization in the neonatal intensive care unit, breastfeeding, food fattiness, and mental health-related issues); (ii) childhood diseases before the IBD diagnosis (e.g., asthma, atopy, hay fever, lactose intolerance, and food allergies), surgery (e.g., tonsillectomy and appendectomy), regular medications, and X-ray diagnoses; (iii) family smoking habits; (iv) childhood lifestyle before the IBD diagnosis; and (v) dietary habits from infancy to childhood.

### 2.3. Measurement

Active and passive smoking were assessed for each family member (i.e., father, mother, sibling [s], and grandparent [s]) separately by the following binary questions: “Did the person have a smoking habit”? and “Did the person smoke in front of the child”? Moreover, the number of cigarettes smoked was assessed by the following question: “How many cigarettes did the person smoke in a day”? In the dose-response model as described below, if the answer to the question “Did the person smoke in front of the child?” was yes, the increasing number of cigarettes smoked per day of the family member indicated a higher passive smoking dose level of the subject.

### 2.4. Statistical Analysis

To investigate the influence of familial smoking on the child’s risk of developing CD, category (iii) of the questionnaire was analyzed for 22 CD patients and 135 controls. In fact, six control subjects were excluded due to either gastrointestinal disorders (i.e., irritable bowel syndrome, invagination, and intestinal obstruction), autoimmune disorders (i.e., Sjögren syndrome), or intractable diseases (i.e., phenylketonuria and Kawasaki disease). Besides, 6 CD cases and 15 controls were excluded due to the lack of information concerning the active smoking status of all family members; moreover, two CD cases and eight controls were also excluded due to the lack of information concerning the number of cigarettes smoked by all active smoking family members. Nevertheless, all the CD cases and controls who had not provided the aforementioned pieces of information were included, after having assumed the missing values being either zero or negative from the negative response to the active smoking status. Furthermore, for simplicity reasons, all the UC cases were excluded in order to focus solely on the contribution from the inhalation pathway; the relationship between tobacco exposure and pediatric UC will be discussed elsewhere via an ingestion pathway-related hypothesis.

Univariable associations between family smoking and pediatric CD were compared using Fisher’s exact test and *t*-test for the categorical and quantitative variables, respectively. A *p*-value < 0.05 was considered statistically significant. Logistic regression analysis was used to estimate the odds ratios (ORs) and 95% confidence intervals (CIs).

To assess the influence of familial passive smoking, we assumed a dose–response relationship between the inhalation intake of passive smoke and the development of pediatric CD. Generally, the inhalation intake of passive smoke (D) is proportional to the child’s peripheral air concentration of tobacco smoke ($C_{A}$), which can be expressed by the following equation [19]:

(1) $$D=B\tau C_{A}$$

where B is the child’s breathing rate and τ is the exposure duration. We assumed that the child’s peripheral air concentration of tobacco smoke is proportional to the number of cigarettes smoked in front of the child, so that the child’s peripheral air concentration of tobacco smoke can be written as follows:

(2) $$C_{A}=a\underset{j}{\sum }n_{j}$$

where j denotes a family member (father = 1, mother = 2, sibling = 3, and grandparent = 4), $n_{j}$ is the number of cigarettes smoked in front of the child, and a is a proportionality factor. We also assumed that the number of cigarettes smoked in front of the child is proportional to the number of cigarettes smoked in a day $N_{j}$, so that the Equation (2) can be rewritten as follows:

(3) $$C_{A}=\underset{j}{\sum }\alpha_{j}N_{j}SHS_{j}$$

where $\alpha_{j}$ is the redefined proportionality factor for the family member j, and $SHS_{j}$ is the answer to the question related to passive smoking (Yes = 1 or No = 0). Consequently, the dose–response relationship model in this study can be expressed as follows [20,21]:

(4) $$\ln\left(\frac{p\left(D\right)}{1-p\left(D\right)}\right)=\beta_{0}+\beta_{1}D=\beta_{0}+\beta_{1}B\tau \underset{j}{\sum }\alpha_{j}N_{j}SHS_{j}$$

where p(D) is the probability of developing pediatric CD, and $\beta_{0}$ and $\beta_{1}$ are the regression coefficients. Furthermore, since the child’s breathing rate and exposure duration vary among different individuals, we assumed that the mean values of those could be determined; as a result, the Equation (4) can be expressed more simply as follows:

(5) $$\ln\left(\frac{p\left(D\right)}{1-p\left(D\right)}\right)=\beta_{0}+\underset{j}{\sum }\beta_{j}N_{j}SHS_{j}$$

As described later, the sex-adjusted model can be expressed by the following equation:

(6) $$\ln\left(\frac{p\left(D\right)}{1-p\left(D\right)}\right)=\beta_{0}+\beta_{1}N_{1}SHS_{1}+\beta_{2}N_{2}SHS_{2}+\beta_{3}N_{3}SHS_{3}+\beta_{4}N_{4}SHS_{4}+\beta_{5}Sex$$

All statistical analyses were performed using IBM SPSS Statistics V25 (IBM Corp., Armonk, NY, USA).

As a sensitivity analysis, multiple imputation analysis was performed using expectation maximization (EM) with the bootstrapping method in order to deal with missing data. Further, to perform the dose–response relationship model calculations in R, we defined the product $N_{j}SHS_{j}$ as a new variable—Defined hereafter as passive smoking from each family member (cigarettes in a day)—In the dose–response models. The missing values of passive smoking were imputed under a missing at random assumption. Since the $N_{j}SHS_{j}$ values are not negative integers, a log–linear transformation could be implemented. We independently analyzed 10 EM imputed after bootstrapped datasets in the dose–response models described in the Equations (5) and (6). These analyses were performed using EZR v1.40, a graphical user interface for R v3.5.2 [22]. Further, the Amelia II package [23] was used for multiple imputations; we averaged the estimates of the variables to a single mean estimate and, subsequently, adjusted standard errors according to Rubin’s rules, by using mice adds and mice packages in R [24,25].

### 2.5. Ethical Considerations

This study was approved by the institutional review board of the Dokkyo Medical University (No. dmu27008), as well as by the local ethics committees of each involved hospital. Written informed consent was obtained from all patients and controls. 

## 3. Results

Regarding the characteristics of the 22 CD patients and 135 controls, there were no significant differences in either the age at recruitment, number of active smokers in the family, or number of persons causing passive smoking; in contrast, a significant difference was detected in terms of sex (*p* = 0.039) (Table 1). The median age at CD diagnosis was 12 years (range 0–14). Besides, one or more smokers smoked in front of the child—The definition of “passive smoking” in this study—More frequently in the control group than in the CD group.

Table 2 shows the results of the univariable analyses of active and passive smoking as well as the number of cigarettes smoked in a day by each family member. Albeit no significant association was found, maternal active and passive smoking seemed to be related to an increased risk of pediatric CD (active smoking: OR, 1.87; 95% CI, 0.69–5.03; passive smoking: OR, 2.09; 95% CI, 0.61–7.10). The frequency of a smoking father was about twice that of a smoking mother or grandparent. The ratios were nearly identical between the CD and control groups. The number of cigarettes smoked by the father was much higher than the number smoked by the mother or the grandparent. However, there was no significant association between paternal smoking and the risk of pediatric CD. The presence of a smoking sibling was extremely low, i.e., only four in the control group were observed.

Table 3 indicates the results of the dose–response relationship model. Passive smoking from the mother was significantly associated with pediatric CD (OR, 1.17 [cigarettes/day]; 95% CI, [1.04–1.31]), even after sex-adjustment (adjusted OR, 1.16 [cigarettes/day]; 95% CI, [1.04–1.30]); besides this, sex did not remain significant in the multivariable dose–response model (adjusted OR, 2.62; 95% CI, [0.97–7.10]). Furthermore, the results of the Hosmer–Lemeshow test were not statistically significant either in the unadjusted (χ^2^ = 1.21, *p* = 0.876) or sex-adjusted (χ^2^ = 3.03, *p* = 0.805) dose–response models.

Table 4 shows the results of sensitivity analysis. Passive smoking from the mother was significantly associated with pediatric CD (OR, 1.10 [cigarettes/day]; 95% CI, [1.01–1.20]), even following sex-adjustment (adjusted OR, 1.10 [cigarettes/day]; 95% CI, [1.00–1.20]). Sex remained significant in the multiple imputation analysis using EM with the bootstrapping method unlike the listwise deletion results in Table 3.

## 4. Discussion

The present study identified an association between passive smoking from the mother and the development of pediatric CD. Although the answers to the binary questionnaires failed to reveal any significant associations between passive smoking and pediatric CD, the results from the dose–response model support our hypothesis that passive smoking is a risk factor for the development of Japanese pediatric CD.

Notably, pieces of evidence that passive smoking increases the risk of pediatric or adolescent-onset CD are being gradually accumulated, although yet not conclusive. A previous age- and sex-matched case–control study reported an association between passive smoking and an increased risk of pediatric CD with a dose–response effect [14]. Similarly, in the present study, a dose–response relationship between passive smoking from the mother and the development of pediatric CD was observed. Furthermore, with regards to Japanese pediatric CD, a significant difference in sex distribution (i.e., male-to-female ratio = 1.8 [1266/689]) was previously reported [26]; consistently, the male-to-female ratio in the present study was 2.1 (15/7).

Remarkably, passive smoking from one or more family members was inversely associated with pediatric CD (Table 1); the same trends were seen also with regards to passive smoking from the father or grandparent (Table 2). A recent Japanese case–control study only reported a positive significant association between passive smoking and CD [17]; in fact, these results seem to be inconsistent with those of the present study. In Japan, among smokers, the percentage of fathers and mothers who smoked indoors were 57% (= 16,131/28,314) and 70% (= 5379/7642), respectively, in 2001 [27], and 36% (= 295/822) and 64% (= 527/823), respectively, in 2014 [28]. As shown in Table 2, among the smokers, the percentage of fathers who smoked in front of the children in the CD and control groups was 31% and 58%, respectively, consistent with the aforementioned indoor smoking studies; however, the value in the control group (58%) is much larger than that of the CD group both in this study (31%) and in the study from 2014 (36%). On the other hand, the percentages of mothers who smoked in front of the children among the smoking mothers were 57% in the CD group and 48% in the control group, thus in good agreement with each other as well as with the previous indoor smoking studies (70% and 64%). A possible explanation could be that the statistical fluctuation in the present study, due to its small sample, might have resulted in an increased percentage of fathers who smoked in front of the children in the control subjects among the smokers. Besides, we did not have information on how many families lived with their grandparents, which may have additionally influenced the present results. Consequently, we introduced the dose–response relationship models to thoroughly investigate the effects of passive smoking on the development of pediatric CD.

Importantly, the effect of passive smoking is directly associated with its inhalation intake yet not with behavioral aspects, such as smoking in front of the child; therefore, our dose–response relationship models described in the Equations (5) and (6) could accurately assess the effect of passive smoking. In such models, the inhalation intake of passive smoking was estimated by using the child’s peripheral air concentration of tobacco smoking. Nonetheless, the information of the concentration of tobacco smoke in the air was never available; thus, we needed to estimate it using the number of cigarettes actively smoked in a day by each family member, by introducing proportionality factors ($\alpha_{j}$). As a result, ORs smaller than 1 reflected a weaker contribution to the contamination with tobacco smoking of the child’s peripheral air. As shown in Table 3, only passive smoking from the mother is a statistically significant factor, with an OR larger than 1. This result indicates that passive smoking from the mother was predominantly associated with the development of pediatric CD, as the number of actively smoked cigarettes by mothers habitually smoking in front of their child was significantly proportional to the risk of pediatric CD. Based on common sense, the mother spends more time with her child than the father. Indeed, Hsin and Felfe reported that the time spent by the child with the mother was about 1.29–2.03 times longer than the time spent with the father [29]. As described above, among smokers, the percentage of mothers smoking indoors was much higher than that of fathers [27,28]. Accordingly, the present results could be explainable based on the senses, so that we can conclude that passive smoking from the mother is one of the most relevant factors associated with the risk of developing pediatric CD.

Listwise deletion was performed in the dose–response relationship models: as a result, we excluded about 8 CD cases (27%) and 23 control subjects (15%). Therefore, as a sensitivity analysis, we performed multiple imputation analyses under missing at random assumption. As seen in Table 4, passive smoking from the mother was still significantly associated with pediatric CD. Consequently, listwise deletion would be rationalized, and we adopted its results as the results of the present study. 

Nevertheless, this study has some limitations. Firstly, since the study sample was rather small, its results may not be strongly inferable. However, the main aim of this study was to verify the association between passive smoking and the risk of pediatric CD. In fact, passive smoking from the mother was significantly associated with pediatric CD in the dose–response relationship models; therefore, maternal smoking cessation is an essential preventive intervention for pediatric CD. Secondly, since this was a retrospective study, the possibility of recall bias has to be considered. However, as smoking is one of the common and important events, the present results may be valuable for further studies of passive smoking and pediatric CD. Thirdly, the exposure dose of passive smoking was only estimated indirectly by using the number of actively smoked cigarettes. Because of the still low incidence rate of pediatric CD in Japan, direct measurements of passive smoking exposure are technically not feasible. However, follow-up studies would still be necessary. Fourthly, approximately 50% of the control subjects were university or college students. However, since university and college students in urban areas in Japan generally come from various places, the residential area might not be an issue. Fifthly, our passive smoking definition was limited to smoking in front of the child; in contrast, in a previous study, passive smoking was defined as smoking at least five cigarettes per day by a parent or sibling who lived in the same house with a case or control subject at the time of symptom onset in the study [14]. Thus, our relatively loose definition of passive smoking in the binary questionnaires may be the cause of no significant differences in passive smoking from the mother. However, as discussed before, our dose–response relationship models yielded results consistent therewith.

## 5. Conclusions

In conclusion, the present study revealed that passive smoking from the mother might be associated with the risk of developing pediatric CD, hence the preventive role of maternal smoking cessation. Nonetheless, follow-up studies comprising environmental measurements of passive smoking exposure doses and genetic factors interaction analysis are still necessary.

## Figures and Tables

**Table 1:** General characteristics of the study participants analyzed in CD cases and controls.

Characteristics	CD (*n* = 22)	Controls (*n* = 135)	*p*-Value ^1^
Sex, *n* (%)			
Male	15 (68.2)	59 (43.7)	**0.039**
Female	7 (31.8)	76 (56.3)	
Age at recruitment, years			
Mean (SD)	14.4 (3.5)	15.6 (4.0)	0.166
Median (Range)	14.5 (6–22)	18 (4–24)	
Age at diagnosis, years			
Mean (SD)	10.8 (3.8)		
Median (Range)	12 (0–14)		
Family smoking, *n* (%)			
One or more smokers	17 (77.3)	100 (74.1)	1.000
No smoker	5 (22.7)	35 (25.9)	
Smoking in front of the child, *n* (%)			
Yes (one or more smokers)	5 (22.7)	61 (45.2)	0.062
No	17 (77.3)	74 (54.8)	

CD, Crohn’s disease; SD, standard deviation. ^1^ *p*-value from Fisher’s exact test (sex, smoking) or *t*-test (age).

**Table 2:** Comparison of active and passive smoking and number of cigarettes smoked by each family member in pediatric CD compared with controls: Univariable analysis.

	CD (*n* = 22)	Controls (*n* = 135)	*p*-Value ^1^	OR (95% CI)
Smoking, *n* (%)				
Father	13 (59.1)	86 (63.7)	0.812	0.82 (0.33–2.06)
Mother	7 (31.8)	27 (20.0)	0.263	1.87 (0.69–5.03)
Sibling	0 (0.0)	4 (3.0)	1.000	–
Grandparent	7 (31.8)	38 (28.1)	0.800	1.19 (0.45–3.15)
Smoking in front of the child, *n* (%)				
Father	4 (18.2)	50 (37.0)	0.095	0.38 (0.12–1.18)
Mother	4 (18.2)	13 (9.6)	0.263	2.09 (0.61–7.10)
Sibling	0 (0.0)	1 (0.7)	1.000	–
Grandparent	1 (4.5)	19 (14.1)	0.312	0.29 (0.04–2.29)
Number of cigarettes smoked, cigarettes/day (SD)				
Father	10.1 (11.1)	10.4 (9.6)	0.905	1.00 (0.95–1.04)
Mother	4.0 (7.8)	2.4 (5.3)	0.253	1.04 (0.97–1.11)
Sibling	0.0 (0.0)	0.3 (2.0)	0.506	–
Grandparent	5.0 (8.6)	5.1 (11.0)	0.952	1.00 (0.96–1.04)

CD, Crohn’s disease; OR, odds ratio; CI, confidence interval; SD, standard deviation; ^1^ *p*-value from Fisher’s exact test (active smoking, smoking in front of the child) or *t*-test (number of cigarettes smoked).

**Table 3:** Crude and multivariable odds ratios and 95% confidence intervals in the dose–response relationship models between pediatric CD and passive smoking.

	Crude OR (95% CI)	Multivariable OR (95% CI)
Source of passive smoking (cigarettes/day)		
Father	0.92 (0.85–1.01)	0.92 (0.84–1.00)
Mother	**1.17 (1.04–1.31)**	**1.16 (1.04–1.30)**
Sibling	–	–
Grandparent	0.89 (0.75–1.05)	0.89 (0.75–1.06)
Sex		
Male	–	2.62 (0.97–7.10)

CD, Crohn’s disease; OR, odds ratio; CI, confidence interval; Numbers in bold font indicate OR increases found to be significant.

**Table 4:** Sensitivity analyses of the dose–response models between pediatric CD and passive smoking.

	Crude OR (95% CI)	Multivariable OR (95% CI)
Source of passive smoking (cigarettes/day)		
Father	0.99 (0.94–1.03)	0.98 (0.93–1.03)
Mother	**1.10 (1.01–1.20)**	**1.10 (1.00–1.20)**
Sibling	1.06 (0.68–1.66)	1.10 (0.67–1.79)
Grandparent	0.91 (0.80–1.03)	0.92 (0.81–1.04)
Sex		
Male	–	**3.52 (1.44–8.59)**

CD, Crohn’s disease; OR, odds ratio; CI, confidence interval; Numbers in bold font indicate OR increases found to be significant.
